# Grape Pomace: Antioxidant Activity, Potential Effect Against Hypertension and Metabolites Characterization after Intake

**DOI:** 10.3390/diseases6030060

**Published:** 2018-07-06

**Authors:** Zuriñe Rasines-Perea, Isabelle Ky, Gérard Cros, Alan Crozier, Pierre-Louis Teissedre

**Affiliations:** 1 Univ Bordeaux, Unité de recherche Œnologie, EA 4577, USC 1366 INRA, ISVV, 33882 Villenave d’Ornon CEDEX, France; zrasines@hotmail.es (Z.R.-P.); isabelleky@gmail.com (I.K.); 2 Institut des Biomolécules Max Mousseron (IBMM), UMR CNRS-5247, Universités Montpellier 1 et 2, Ecole Nationale Supérieure de Chimie de Montpellier, BP 14491, 34093 Montpellier CEDEX 5, France; gerard.cros@umontpellier.fr; 3 Department of Nutrition, University of California, Davis, CA 95616, USA; alan.crozier44@gmail.com

**Keywords:** grape pomace, polyphenols, hypertension, metabolites characterization

## Abstract

Observational studies indicate that the intake of polyphenol-rich foods improves vascular health, thereby significantly reducing the risk of hypertension and cardiovascular disease (CVD). Therefore, the aim of this study was to analyse the remained potential of grape by-products from important Rhône Valley red wine cultivars: Grenache, Syrah, Carignan, Mourvèdre and Alicante. For that, six different extracts from grape pomaces, selected by their antioxidant activity, were studied in vivo during six weeks with spontaneously hypertensive rats (SHR). Extracts used in SHR1, SHR2 and SHR6 groups presented a « rebound effect » on systolic blood pressure, whereas the other extracts do not change it significantly. The bioavailability of Grenache (GRE1) (EA70) seed pomace extract (SHR1 group), Mouvendre (MOU) (EA70) skin pomace extract (SHR5 group) and Alicante (ALI) (EA70) skin pomace extract (SHR6 group) was studied by High Performance Liquid Chromatography with Photodiode Array detector and Electrospray Ionization Mass Spectrometer (HPLC-PDA-ESI-MS^n^) in urine, plasma and tissues to search differences on the metabolism of the different extracts intake.

## 1. Introduction

Polyphenols are the most abundant and ubiquitous secondary metabolites present in the plant kingdom with more than 8000 phenolic structures currently known. These compounds play an important role in plant growth and reproduction, providing protection against biotic and abiotic stress such as pathogen and insect attack, ultra violet (UV) radiation and wounding [1,2].

Dietary intake of (poly)phenols has been estimated to be about 1 g/day [3]. Their intake is 10 times greater than that of vitamin C and 100 times that of vitamin E or the carotenoids [4]. As a result, phenolic compounds are currently receiving much attention because of their favourable health effect related to their antioxidant properties. Indeed, several studies have focused their attention on the components of red wine (mainly polyphenols and especially resveratrol) since the so-called “French paradox” was first described [5] in order to explain the relationship observed between wine consumption and the incidence of cardiovascular disease (CVD). There is extensive evidence to support the influence of wine intake on cardiovascular health [6,7,8,9], controversy remains whether red wine in particular exerts beneficial effects compared with other alcoholic beverages [10,11] or simply alleviates the detrimental influence of alcohol on blood pressure (BP) [12,13]. No changes in BP were observed in acute studies with healthy volunteers but an increase in the heart rate was reported after red wine consumption [14,15], whereas in coronary artery disease (CAD) patients, a decrease in systolic and diastolic BP was noted together with an increase in heart rate, 1 h post wine (red and white) intake [16]. Other studies have reported no changes in hemodynamics or BP after medium-term daily intake of either red wine or its dealcoholized equivalent [17,18].

Overall results of polyphenols consumption may be promising but they remain inconclusive and further prospective studies assessing dietary polyphenol exposure and studies using other methods to evaluate exposure (i.e., markers of consumption, metabolism, excretion) have been recommended, as concluded in a recent meta-analysis summarizing [19]. A recent systematic review using intervention studies confirmed that urinary polyphenol metabolites could serve as dietary biomarkers with high recovery yields and high correlations with intakes of polyphenol-rich food [20].

In this article, different grape pomace extracts were studied for their possible influence against hypertension disease, as they present an important under used residue of the wine making process. A large number of publications evidenced the abundance quantity of polyphenols in grape seeds and skins, showing significant antioxidant capacity. It is therefore obvious that it is a natural source of polyphenols that accounts for about 20% of grapes weight used to make wine [21,22]. Finally, some metabolites in urine, plasma and tissues were measured for accurate and precise estimation of dietary exposures and possible differences between the grape pomace extracts

## 2. Materials and Methods

### 2.1. Chemicals

Deionized water was purified with a Milli-Q water system (Millipore, Bedford, MA, USA). High Performance Liquid Chromatography (HPLC) grade acetonitrile (HPLC ≥ 99%), ethyl acetate (HPLC ≥ 99%), methanol (HPLC ≥ 99%), ethanol (HPLC ≥ 99%) and acetone purchased from Scharlau (Sentmenat, Barcelona, Spain). The following chemicals were obtained from Sigma Aldrich (Saint Louis, MO, USA): (+)-catechin (≥98%), (−)-epicatechin (≥98%), B_1_ [(−)-epicatechin-(4β-8)-(+)catechin] (≥98%), procyanidin dimer B_2_ [(−)-epicatechin-(4β-8)-(−)-epicatechin] (≥98%), cyanidin-3-*O*-glucoside chloride (≥98%), delphinidin-3-*O*-glucoside chloride (≥98%), malvidin-3-*O*-glucoside chloride (≥98%), peonidin-3-*O*-glucoside chloride (≥98%), gallic acid (≥98%), 2,2-Diphenyl-1-picrylhydrazyl (DPPH), 6-hydroxy-2,5,7,8tetramethylchroman-2-carboxylic acid (Trolox) (≥97%), 2,2'-azinobis(3ethylbenzothiazoline-6-sulfonic acid) diammonium salt (ABTS) (≥98%), potassium persulfate (≥99%), fluorescein (≥98%), 2,2′-azobis (2-methylpropionamidine) dihydrochloride (AAPH) (≥97%), sodium dihydrogen phosphate dehydrate (≥ 98%), disodium hydrogen phosphate dodecahydrate, 2,4,6-tri(2-pyridyl)-s-triazine (TPTZ) (≥98%), iron (III) chloride hexa-hydrate (≥98%), iron (II) sulphate hepta-hydrate (≥98%), l-ascorbic acid (≥99%), derivatization reagent (pyridine (≥99.8%) and *N*-methyl-*N*(trimethylsilyl)trifluoroacetamide [MSTFA] (≥98.5%)) and *N*,*N*-dimethyl formamide (DMF) (≥99.8). The Laboratory of Organic Chemistry and Organometallic (Université Bordeaux 1) synthesized procyanidins dimers B_3_ [(+)-catechin-(4α-8)-(+)-catechin] and B_4_ [(+)-catechin-(4α-8)-(−)-epicatechin] and a trimer (T) [(+)-catechin-(4β-8)-(+)-catechin-(4β-8)-(−)-epicatechin] [23].

Ferulic acid (≥99%), was obtained from AASC Chemicals (Southampton, U.K.). EDTA and pentobarbital solution used in rats feeding studies were supplied by Sigma Aldrich. Hydrochloric acid (37%), sodium hydroxide (≥8%), acetic acid (≥99%), phosphoric acid (≥85%) and formic acid (≥95%), were purchased from Fisher Scientific Ltd. (Loughborough, Leicestershire, UK).

### 2.2. Plant Materials

This study was conducted with 2010 grapes pomaces from *V. vinifera* L. cv. Grenache (from two different locations [GRE1 and GRE2]), Syrah (from two different locations [SYR1 and SYR2]), Carignan (CAR), Mourvèdre (MOU), Counoise (COU) and Alicante (ALI), provided by the Château de Beaucastel located in the Vallée du Rhône, appellation of Châteauneuf-du-Pape. Details are provided below.

Samples were selected on the basis of their high content of polyphenols according to previous studies [24].

### 2.3. Animals for In Vivo Experiences

For in vivo experiments, spontaneously hypertensive rats (SHR) and normotensive control Wistar-Kyoto (WKY) rats were purchased from Laboratoire Janvier (Le Genest St. isle, France). Animals were maintained at a temperature of 23°C with 12 h light/dark cycles. Tap water and standard diet A 04 SAFE (Augy, France) composed of 83.9% of cereals and cereal by-products, 8.0% of vegetable proteins (soya bean meal, yeast), 4.1% of vitamin and mineral mixtures and 4% of animal proteins (fish) were given ad libitum. They were daily treated with grape pomace extract by gavage during 6 weeks at a dose of 21 mg/kg/day. Rats were divided in different groups: control group (6 WKY), SHR control group (5 SHR treated with 3% EtOH) and 6 groups of 4 SHR rats treated with pomace extract dissolved in 3% EtOH (SHR1: Grenache seed pomace extract, SHR2: Syrah seed pomace extract, SHR3: Syrah skin pomace extract, SHR4: Carignan seed pomace extract, SHR5: Mourvèdre skin pomace extract and SHR6: Alicante skin pomace extract). Blood pressure was measured by the tail-cuff method. The average of three pressure readings was recorded for each measurement.

Food intake and body weight of animals were recorded once a week. Prior to each experiment, adaptation period was allowed. Experiments were performed following European Community animal experiments ethical regulations.

The animal protocols used in this work were evaluated and approved by the Animal Use and Ethic Committee of Languedoc Roussillon Montpellier (reference CEEA-LR-13012) approved on 11 July 2013 and valid for 5 years. They are in accordance with European guidelines and French law for Laboratory Animal Experimentation.

### 2.4. Samples Preparation

#### 2.4.1. Grape Pomace Samples Preparation

One hundred grams of GRE1, SYR1 and CAR seeds and GRE2, SYR1, SYR2, CAR, MOU and ALI skins were extracted in triplicate using 350 mL of distilled water for 1 h under magnetic agitation at 50 °C. In parallel, under the same conditions, these samples were also extracted using a 70% hydro-alcoholic solution (70:30, Ethanol:Water, *v*/*v*) solution. The centrifugal supernatants were evaporated and lyophilized to obtain two types of samples: an aqueous sample (EAQ) and a 70% hydro-alcoholic sample (EA70) for each variety and part.

#### 2.4.2. Sample Collection from In Vivo Experiments

Urine was collected at two time points (0–8 h and 8–24 h) at day 1 and day 7 of gavage from metabolic cage in falcon tubes and immediately stored at −80 °C. At day 7, four hours after gavage, rats were anaesthetized with lethal dose of pentobarbital (60 g/L pentobarbital, 60 mg/kg body weight) and sacrificed by decapitation. Blood was drawn in an EDTA-moistened tube. Tubes were centrifuged at 2300× *g* for 10 min at 4 °C. Plasma was separated from erythrocytes before being stored at −80 °C. Tissues including heart, liver and kidneys were collected, rinsed, weighted, grounded to a powder and stored at −80 °C along with plasmas and urines prior to analysis.

### 2.5. Analytical Methods

#### 2.5.1. Antioxidant Assays

The antioxidant capacity of pomace extracts was assessed by ABTS radical cation (ABTS^•+^), DPPH, Ferric Reducing Antioxidant Potential (FRAP) and Oxygen Radical Absorbance Capacity (ORAC) test as it is described in previous studies [25].

#### 2.5.2. Blood Pressure Measurement

Blood pressure was followed and accessed by tail-cuff method with a LETICA LE 5002 Scientific Instrument electrosphygomanometer (Panlab, Barcelona, Spain) under conscious condition, in a calmed and regulated between 29 °C and 32 °C darkened room. Rats were trained to the measurements, handled with care and covered with a fabric during the record in order to minimize stress. In this method, the reappearance of pulsation on a digital display of the blood pressure cuff is detected by a pressure transducer, amplified and recorded digitally as the systolic blood pressure. The average of the three pressure readings was recorded if only the difference between two measurements was below 20 mmHg.

#### 2.5.3. Extraction of Phenolics from Tissues

Rat tissues from the same experimental group were pooled together in equal proportions. To 60 mg to freeze-dried tissues were added 50 µL of ascorbic acid 1% and 100 µL of phosphoric acid 4%. Each sample was spiked with 1 µg of ethyl gallate as an internal standard. The samples were first extracted with 800 µL of water/methanol/phosphoric acid 4% (94/4.5/1.5, *v*/*v*/*v*) using a sonicator for 30 s (Digital Sonifier^®^ model S-150D ultrasonic cell disruptor, Branson, Teltow, Germany) and maintained in ice to avoid heat. Samples were then centrifuged for 15 min at 16,100× *g*, 20 °C in a 0.2 µm Micro-Spin^TM^ Eppendorf filter (Alltech Associates Applied Sciences, Lancashire, UK). The supernatant was decanted and the pellet re-extracted three more times with 500 µL of the same solvent as described above, after which it was centrifuged. The four supernatants were combined. A mixture of 1 mL of the assembled supernatant and 1 mL of phosphoric acid 4% was loaded onto OASIS^®^ HLB cartridges (3cc, 60 mg) previously conditioned with 1 mL of methanol and 1 mL of 0.2% acetic acid. Column was washed with 1 mL of phosphoric acid 4% and with 1 mL of 0.2% acetic acid. The retained compounds were eluted with 2 × 1 mL acetone/Milli-Q water/acetic acid solution (70/29.5/0.5, *v*/*v*/*v*).

The eluate was reduced to dryness using a Speedvac concentrator (SPS SpeedVac, Thermo Savant, Waltham, MA, USA) and resuspended in 25 µL of acidified methanol (1% acid formic) to which was added 225 µL of 0.1% aqueous formic acid. Once resuspended, extracts were centrifuged at 16,100× *g* for 10 min at 4 °C in a 0.2 µm Micro-Spin^TM^ Eppendorf filter (Alltech Associates Applied Sciences, Lancashire, UK) prior to analysis by HPLC-PDA-MS^n^.

#### 2.5.4. Extraction of Phenolics from Plasmas

Rat plasmas from the same group were pooled together in equal proportions before extraction [26]. Plasma samples were defrosted and spiked with 1 µg of ethyl gallate as an internal standard. Plasma was added drop wise while vortex to a 15 mL falcon tube containing 3.4% phosphoric acid and kept on a freeze water bath. Sample was then loaded onto OASIS^®^ HLB cartridge (3 mL, 60 mg) previously conditioned with 1 mL of *N*,*N-*dimethyl formamide (DMF)/methanol (7/3, *v*/*v*) and 0.5% (*v*/*v*) acetic acid in water. Cartridge was washed with 3 mL of 0.5% acetic acid in water (*v*/*v*) and 1 mL of water/methanol/acetic acid (80/20/0.5). For elution, cartridge was dried and eluted with 2 × 1 mL of DMF/methanol (7/3, *v*/*v*). The eluate was collected in a tube containing 200 µL of 0.5% (*v*/*v*) acetic acid in methanol and reduced to approximately 50 µL using a Speedvac concentrator (SPS SpeedVac, Thermo Savant, Waltham, MA, USA). Sample was then resuspended in 25 µL of acidified methanol (1% acid formic) to which was added 225 µL of 0.1% aqueous formic acid. Extract was then centrifuged at 16,100× *g* for 10 min at 4 °C prior to analysis by HPLC-PDA-MS^n^ within 24 h.

#### 2.5.5. Urine Analysis

Urine samples were defrosted, vortexed and centrifuged at 16,100× *g* for 10 min at 4°C prior to the analysis by HPLC-PDA-MS^n^.

#### 2.5.6. HPLC-PDA-ESI-MS^n^ Analysis of Procyanidin Metabolites

Quantification of metabolites in urines, plasma and tissues was carried out using two Surveyor HPLC systems, both equipped with sampler cooler maintained at 4 °C and a PDA detector. One of them was equipped with a Finnigan LCQ Duo ion trap mass spectrometer for urine analysis. The second one equipped with a LCQ Advantage ion trap mass spectrometer for tissue and plasma analysis.

Mass spectrometers were both fitted with an electrospray interface (ESI) (Thermo Fisher scientific, San Jose, CA, USA). Separation was performed on a 250 × 4.6 mm i.d. 5 μm Kinetex phenyl-hexyl 100 Å column (Phenomenex, Macclesfield, U.K.), maintained at 40 °C. The mobile phase was pumped at a flow rate of 1 mL/min with a gradient over 30 min of 5–60% methanol in 0.1% aqueous formic acid (analysis of urines) or a gradient over 30 min of 5–65% in 0.1% aqueous formic acid (analysis of tissues). The column eluate initially passed through the PDA detector and was then split, with 0.2 mL/min directed to the mass spectrometer fitted with an electrospray interface operating in negative ion mode. The tuning of the mass spectrometer was optimized by infusing a standard of (−)-epicatechin, dissolved in the initial HPLC mobile phase, into the source at a flow rate of 0.2 mL/min.

Procyanidin metabolites were firstly identified using full-scan data-dependent MS^2^ scanning from *m*/*z* 100 to 700. Compound identities were confirmed by MS^3^ consecutive reaction monitoring with a collision energy set at 30%. Following HPLC separation and MS^3^ identification, flavan-3-ols and their metabolites were quantified using selective reaction monitoring (SRM mode). Quantification of 5-(hydroxyphenyl)-4-hydroxyvaleric acid*-O-*glucuronide (*m*/*z*, 401/225) and 5-(hydroxyphenyl)-γ-valerolactone*-O-*sulphate (*m*/*z*, 287/207) was by reference to the aglycone 5-(3′,4′-dihydroxyphenyl)-γ-valerolactone while 5-(hydroxyphenyl)-4hydroxyvaleric acid*-O-*sulphate (*m*/*z*, 305/225) levels were measured using a calibration curve obtained with 5-(3′,4′-dihydroxyphenyl)-4-hydroxyvaleric acid.

The other metabolites were quantified as (−)-epicatechin equivalents.

#### 2.5.7. Statistical Analyses

All measurements were performed in triplicate. Results are expressed as means ± standard deviation (SD). One-way ANOVA was performed to test the effects of variation factors (different samples) on each variable. If significant effects were found at a 95% confidence interval, ANOVA was followed by a Tukey’s HSD and Duncan post hoc test to identify differences among groups. These analyses were performed using Statistica V.7 Software (Statsoft Inc., Tulsa, OK, USA).

## 3. Results and Discussion

### 3.1. Grape Pomace Seed and Skin Antioxidant Activities Evaluation

The antioxidant potential was determined in order to select the most active grape pomace seeds and skins among the studied varieties. The choice of assay method is often based on speed, simplicity, ease of use and instrumentation availability. More than one type of measurement needs to be performed to take into account the various mode of action of antioxidants [27,28]. In this work, the free radical scavenging potential was evaluated by three spectrophotometric tests: the FRAP, ABTS^•+^ and DPPH and a spectrofluorometric test, the ORAC test.

Concerning seed extracts, the four antioxidant analytical techniques gave the same classification both for EAQ and EA70. The highest antioxidant activities were found in SYR1 for both types of extracts. Results were correlated with previous analyses which evidenced SYR1 as having a substantial amount of flavan-3-ols, procyanidins and anthocyanins. Antioxidant activities of EAQ and EA70 grape pomace seed extracts were shown in Table 1.

In skins, results obtained by the different antioxidant analyses were more disparate, especially in EA70 extracts (Table 2). In aqueous extracts, the highest antioxidant activity was found in SYR1 and ALI. This observation was observed with every test and correlated well with previous results evidencing these extracts as containing high phenolic content. In EA70, different antioxidant tests did not give the same extract classification. Despite this fact, SYR1 skin extract was classified as being the first or second extract showing the highest antioxidant capacity in the four tests (ORAC: 1912.6 µM TE/g DW; FRAP: 1.52 mM Fe^2+^/g DW, ABTS^•+^: 2614.5 µM TE/g DW and DPPH: 1391.7 TE/g DW).

EA70 extracts exhibited higher potential and proved to be more effective than EAQ extracts. Because of bioavailability, metabolism, biotransformation and chemical reactivity, in vitro capacity cannot be simply extrapolated [29]. Therefore, in order to evaluate the health effects of these extracts, in vivo experiments need to be performed and the effects of antioxidant may be evaluated using appropriate biomarkers in biological fluids and tissues.

### 3.2. In Vivo Results

#### 3.2.1. Systolic Blood Pressure Results

Spontaneously hypertensive rats (SHR) selected for this study is frequently used to carry out studies on the antihypertensive effect of functional food ingredients. This strain represents nowadays the best experimental model for essential hypertension in humans and has shown its efficiency in many studies [30,31,32,33,34,35].

In order to evaluate the in vivo effect of grape pomace extracts and their potential effect on hypertension, rats were fed with different grape pomace EA70 extracts at a dose of 21 mg/kg/day, equivalent to a daily dose of 70 kg human consumption of 0.5 L of wine. The study was conducted over six weeks including three weeks of treatments, one week of treatment resumption followed again by two weeks of treatment.

**Figure 1:**
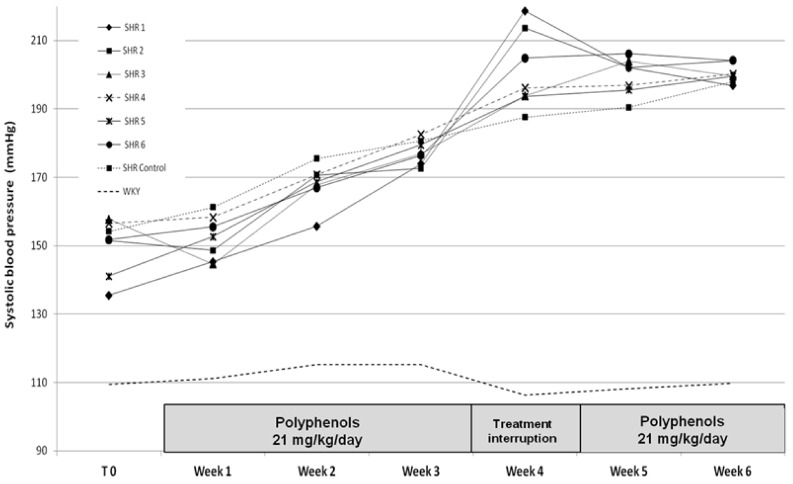
Effect of polyphenolic extracts on the mean systolic blood pressure during the 6-week study.

The mean systolic blood pressure of SHR rats was comprised between150 mmHg at the beginning of the experiment and 190 mmHg after five weeks (Figure 1).

Polyphenolic extracts given to SHR rats seemed to have little effect on systolic blood pressure which increased gradually (except for the SHR1 after 1 week of treatments). However, after three weeks of extracts intake, gavage intolerance was observed and caused difficulty with the administration of polyphenolic extracts, forcing the interruption of the treatment for one week. This treatment interruption was followed by an increase of the systolic blood pressure in SHR1 (Grenache seed pomace extract), SHR2 (Syrah seed pomace extract) and SHR6 (Alicante skin pomace extract) group compared to the SHR control group. This phenomenon can be interpreted as a « rebound effect » commonly observed with anti-hypertensive drugs and may reveal an antihypertensive effect of grape pomace extracts. The treatment resumption at weeks 5 and 6 describe a relative decrease in systolic blood pressure for SHR1 and SHR2 groups and a stabilisation of systolic blood pressure for the SHR6 group, which implies that if the treatment was pursued for a higher period of time, the values of systolic blood pressure may have been improved significantly with respect to the SHR control group.

#### 3.2.2. Quantification of Polyphenolic Metabolites in Urine

Based on previous results, extracts SHR1 (GRE1 (EA70) seed pomace extract), SHR5 (MOU (EA70) skin pomace extract) and SHR6 (ALI (EA70) skin pomace extract) have been chosen to bioavailability study.

Urine samples were collected at 0–8 h and 8–24 h on day 1 and day 7 after the ingestion of different grape pomace extracts. The urine did not contain any of the original grape pomace extract flavan-3-ols and procyanidins but glucuronides and methylglucuronides of (epi)catechin, as well as valerolactone and valeric acid phase II glucuronide and sulphate metabolites were detected. Compared to other studies in which rats were fed grape derived products [36,37,38], sulphate derivatives were found as one of the main metabolites, whereas in this work, sulphates were present in very low amounts. The reason for these varying metabolite profiles, especially the dominance of glucuronides in some studies and sulphates in order could be due to losses of (epi)catechin*-O-*sulphates during sample processing before analysis [39]. The difference in the metabolic profile of (epi)catechins may also be attributed to the different flavan-3-ols composition of the matrix (e.g., grape seed/skin extracts, tea, cocoa).

A total of 18 metabolites were identified in urine. To have an overall picture of metabolite excretion, in this article, the sum of urinary (epi)catechin-*O*-glucuronide, *O*-methyl-(epi)catechin-*O*-glucuronide, 5-(hydroxyphenyl)-γ-valerolactone-*O*-glucuronide, 5-(hydroxyphenyl)-γ-valerolactone-*O*-sulphate, 5hydroxyphenyl-4-hydroxyvaleric acid-*O*-sulphate, 5-(phenyl)-4-hydroxyvaleric acid-*O*sulfate, isoferulic acid-4-*O*-sulphate was calculated. The results are presented in Table 3 and Table 4.

Higher levels of *O-*methyl-(epi)catechin*-O-*glucuronide relative to (epi)catechin*-O-*glucuronide were found in every urine sample. Sulphate derivatives of 5-(hydroxyphenyl)-γ-valerolactone were excreted in greater amounts than glucuronides. A decrease of 5-(hydroxyphenyl)-4-hydroxyvaleric acid-*O-*sulfate from 0–8 h to 8–24 h at day 1 was observed while an increase of 5-(phenyl)-4hydroxyvaleric acid*-O-*sulphate occurred at 8–24 h compared to 0–8 h in all rat urines at day 1 and day 7. A previous report on a human almond skin polyphenol bioavailability [40] suggested that partial dihydroxylation reactions occur gradually as colonic metabolism progresses. These authors observed a change in the hydroxylation pattern of the phenyl ring from di- to mono- and unhydroxylated forms. Dihydroxylated derivatives were found 6–10 h after the intake, monohydroxylated forms were observed at 6–24 h and unhydroxylated derivatives were found 10–24 h after the intake.

**Figure 2:**
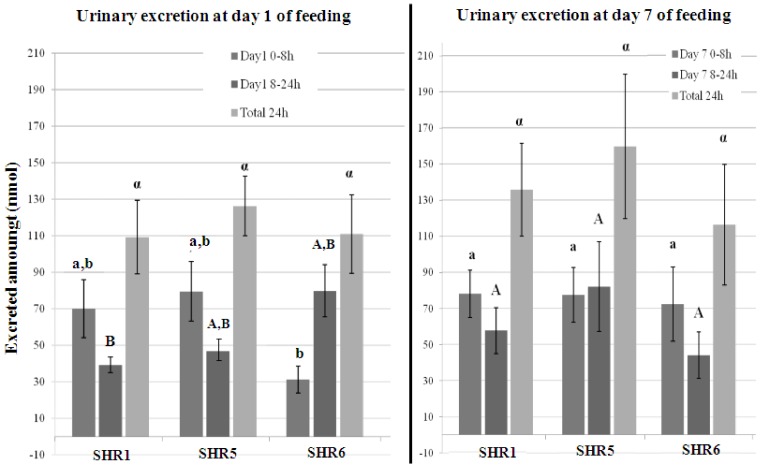
Total urinary metabolites excreted at days 1 and 7, 0–8 h, 8–24 h and 0–24 h after ingestion of grape pomace extracts. SHR rats fed with: SHR1, Grenache (GRE1) EA70 seed pomace extract; SHR5, Mourvèdre (MOU) EA70 skin pomace extract and SHR6, Alicante (ALI) EA70 skin pomace extract. a,A,α,B,b: Anova was performed to compare values between different experimental groups during 0–8 h (small letters), 8–24 h (capital letters) and 24 h (symbol letters) at day 1. Same letters indicate no significant differences between the values (*p <* 0.05). Data are expressed as mean values in nmol ± standard error.

In order to compare efficiency of grape pomace extracts, the sum of metabolites that are excreted in significantly higher amounts compared to their respective control (*p <* 0.05) are presented in Figure 2 for days 1 and 7.

At day 1 of SHR rat gavage, substantial 0–8 h urinary excretion of metabolites was observed in the SHR1 and SHR5 experimental groups unlike SHR6 in which highest excretion occurred in 8–24 h urine. Considering the 0–8 h samples, high urinary excretion was found in E5 (80 ± 16 nmol) followed by E1 (70 ± 16 nmol). During 8–24 h collection period, excretion of metabolites was more pronounced with E6 (80 ± 7 nmol).

At day 7 no significant difference was observed between different experimental groups in both 0–8 h, 8–24 h and even over 0–24 h. This phenomenon can be due to a high absorption and excretion reducing variability between subjects of the same group. Overall, total 24 h urinary excretion at day 7 ranged from 117 ± 33 nmol in SHR6 to 160 ± 40 nmol in SHR5 and was higher than that obtained at day 1 which varied from 109 ± 29 to 127 ± 17 nmol.

The percentage of intake of grape pomace extracts administrated alone or in association with verapamil to SHR rats was calculated for each time point (Table 5).

At day 1, excretion as a percentage of intake varied from 1.02% to 1.57%. Highest recoveries were observed in the SHR5 group (1.57%, over 0–24 h). At day 7, an increase of intake was observed for the two collection periods and, as a consequence, over 0–24 h. The recoveries ranged from 1.43% in SHR6 to 4.41% in SHR5. A large increase in metabolite excretion was observed in SHR5 group with 4.41% of intake at day 7 compared to 1.57% at day 1. These observations suggested that over time, SHR rats may be able to ingest higher doses of polyphenols especially those contained in MOU (EA70) skin pomace extract.

The recoveries obtained in this study were low compared to other studies. For instance, a urinary excretion of intake 0–24 h corresponding to 33% was found after ingestion of a grape seed extract containing a wide array of monomeric to polymeric flavan-3-ols ingestion [36]. In humans, urinary recovery of flavan-3-ols accounted 2–10% for red wine catechins [41]. However, higher amounts of flavan-3-ol monomers and polymers were ingested by humans than rats. A research [42] showed in a green tea flavan-3-ol feeding study with ileostomists that (epi)gallocatechins are subjected to strictly limited absorption whereas (epi)catechins can be readily absorbed even with an increasing dose. Thus, the low number of polyphenols fed in this study compared to other studies could be the cause of these recovery differences.

#### 3.2.3. HPLC-ESI-MS^n^ Analysis of SHR Rat Plasma

SHR rats’ plasma was screened for flavan-3-ol and procyanidin metabolites using, in the first instance, the full-scan MS mode from *m*/*z* 100 to 700 in negative ionization mode. Peak identifications were based on data dependent MS^2^ and on previous studies [36,37,43,44].

A total of 11 metabolites were found in SHR rat plasma collected 4 h after grape pomace extract ingestion. Plasma did not contain any of the original grape pomace extract flavan-3-ols and procyanidins but glucuronide, methyl-glucuronide and di-methylglucuronide of (epi)catechin and glucuronide, sulphate and mono-substituted valerolactone and valeric acid were present. These metabolites were also detected in urine with the exception of the di-methyl-(epi)catechin-*O*-glucuronide. Metabolites were occurred mainly as glucuronides rather than sulphates. These observations were in accordance with a previous study by [36] in which rats were fed with grape seed extracts. These investigators detected only glucuronidated forms of (epi)catechins in plasma while sulphates predominated in urine.

**Figure 3:**
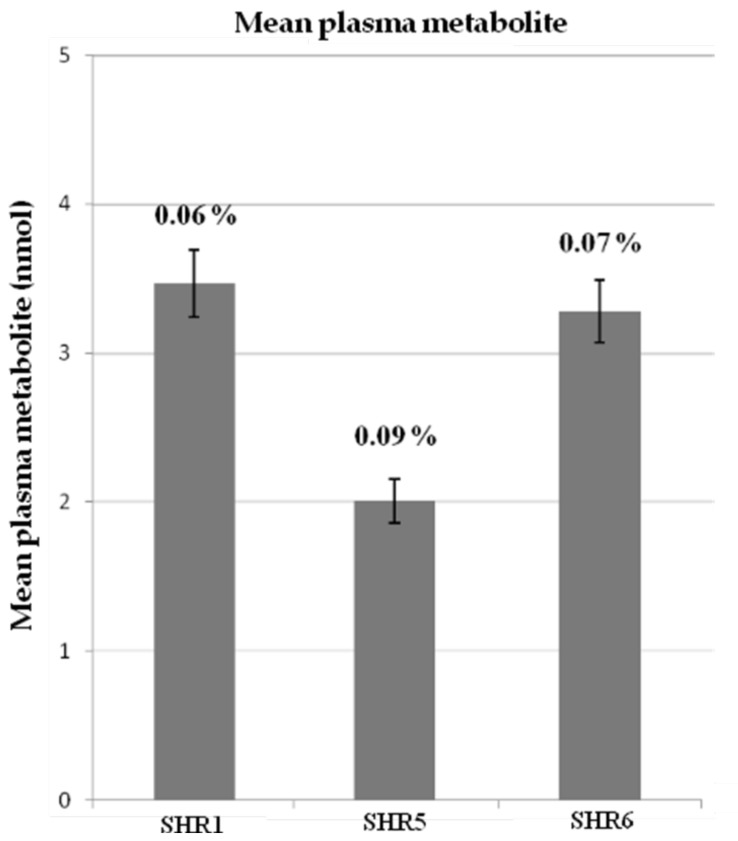
Mean plasma metabolite levels in different experimental group at day 7, 4 h after ingestion of grape pomace extracts *(p <* 0.05*)* and their respective recoveries (%). Data expressed as nmol ± standard deviation. SHR rats fed with: SHR1, Grenache (GRE1) EA70 seed pomace extract; SHR5, Mourvèdre (MOU) EA70 skin pomace extract and SHR6, Alicante (ALI) EA70 skin pomace extract.

Quantitative data are presented in Table 6. Generally, lower amounts of metabolites were observed in plasma than in urine showing that flavan-3-ols are rapidly turned over the circulatory system, and, rather than accumulating, are excreted via the kidneys. Figure 3, summarizes plasma metabolite amounts in different experimental group at day 7, 4 h after grape pomace extracts ingestion *(p <* 0.05*)* and their respective recoveries (%).

SHR5 urinary excretion indicated substantial absorption of polyphenols (4.41% of intake) but a recovery of 0.09% in plasma implies a rapid turnover of polyphenols in the circulatory system. Grape pomace polyphenols were absorbed to a lesser extent by rats fed with extracts of SHR1 and SHR6, as illustrated by low recoveries of metabolites.

#### 3.2.4. HPLC-ESI-MS^n^ Analysis of SHR Rat Tissues (Heart, Liver and Kidneys)

Metabolites in SHR rat tissues were detected using the same procedure as described for urine and plasma. No (epi)catechin conjugates were found but a 5-(hydroxyphenyl)-γ-valerolactone-*O*-glucuronide (*m*/*z* 383/207, 163) and a 5-(hydroxyphenyl)-γ-valerolactone-*O*-sulphate (*m*/*z* 287/207, 163) were detected and quantified by SRM. Their distribution in tissues is presented in Table 7. Data are expressed as nmol per organ ± standard deviation. Both glucuronide and sulphate metabolites of the 5-(hydroxyphenyl)-γ-valerolactone were detected but the glucuronide predominated.

Heart tissues contained more glucuronidated metabolites than sulphated. 5-(hydroxyphenyl)-γ-valerolactone-*O*-sulphate, was detected only in E1 and E5 experimental groups at a level of 0.11 ± 0.00 nmol.

Kidneys contained more 5-(hydroxyphenyl)-γ-valerolactone-*O*-sulphate than the liver.

Liver contained the highest level of metabolites with most present as glucuronides. A 5-(hydroxyphenyl)-γ-valerolactone-*O*-sulphate was only detected in SHR rats fed with ALI (EA70) skin pomace extract + verapamil at 7.24 ± 0.6 nmol. Only SHR from E5, VE5 and VE6 experimental groups contained significantly higher amounts (210 ± 12 nmol, 198 ± 3 nmol and 194 ± 7 nmol, respectively) of 5-(hydroxyphenyl)-γ-valerolactone-*O*glucuronide compared to control groups.

The absorption and arguably metabolism, of flavan-3-ols and procyanidins initially takes place during transfer through the wall of the small intestine after which further phase II metabolism occurs in the liver. As liver appear to be the most important organs involved in flavonoid metabolism, it was not surprising to find such a high level of metabolites. Some of the conjugated metabolites can be actively effluxed back into the lumen of the small intestinal and/or may be transported to other organs through the bloodstream as shown by their presence in the heart. In addition, metabolites were also detected in kidneys no doubt as a consequence of renal excretion. It should be noted that the time of tissue sampling may be of importance and metabolites detection depends on the kinetics of their accumulation and elimination in the tissues. In this study, tissues which were shown to contain metabolites were collected 4 h after the ingestion of grape pomace extracts. A study carried out in 2005 found flavan-3-ol metabolites in the liver of rats 1 h and 4 h after ingestion of a grape seed extract but none were detected 6, 12 and 24 h after intake [36]. This indicates a rapid elimination of flavan-3-ol metabolites in keeping with them being treated as xenobiotics by the body.

## 4. Conclusions

This study confirms that substantial levels of polyphenols after the winemaking process, remain in pomace in quantities sufficient to exert anti-hypertensive effects. In addition, according to the extract used and its composition, it is feasible to modulate anti-hypertensive effects by amplifying or decreasing polyphenols absorption. Therefore, it will be interesting to elucidate the exact mechanisms and compounds involved in this phenomenon in order to have a better control on blood pressure regulation and facilitate the choice of effective grape pomace extracts for further experiments. Moreover, it will be useful to investigate the effect of different flavan-3-ol fractions (i.e., oligomeric, monomeric) and anthocyanin fractions (i.e., glucosides, acetylated glucosides and coumarylic glucosides) in order to identify whether anti-hypertensive effects are linked to a particular compound or to the extracts as a whole.

As SHR represents a good model to investigate hypertension, studies could be extended to human clinical trials. For clinical tests, different parameters have to be taken into account such as the subjects (i.e., pre-hypertensive or hypertensive subjects), the dose used, the diet, biological fluid collections and biological markers to be quantified. In addition, different processes will have to be considered such as the election of grape pomace varieties and their parts (seeds/skins), the extraction processes which will be used, the dosage and the galenic formulation used in order to provide great stabilisation of the active substances.

Moreover, analyses on SHR faeces must be done to complete the polyphenols excretion.

## Figures and Tables

**Table 1:** Antioxidant activity characterization in EAQ and EA70 grape pomace seed extracts.

		ORAC ^2^	FRAP ^2^	ABTS^•+ 2^	DPPH ^2^
**Seeds-EAQ**	**GRE1 ^1^**	1466.4 ± 29.6 ^a^	0.63 ± 0.02 ^a^	1203.2 ± 24.1 ^a^	410.8 ± 43.3 ^a^
	**SYR1 ^1^**	2230.7 ± 101.7 ^b^	1.33 ± 0.08 ^c^	2432.6 ± 56.0 ^c^	1037.1 ± 64.0 ^b^
	**CAR ^1^**	2058.6 ± 85.1 ^b^	1.06 ± 0.08 ^b^	1948.8 ± 61.1 ^b^	1050.6 ± 30.1 ^b^
**Seeds-EA70**	**GRE1 ^1^**	1926.7 ± 108.6 ^a^	1.28 ± 0.01 ^a^	2813.2 ± 90.0 ^a^	1277.6 ± 54.7 ^a^
	**SYR1 ^1^**	2614.0 ± 150.9 ^a^	1.45 ± 0.16 ^a^	3601.2 ± 88.6 ^b^	1685.9 ± 130.7 ^b^
	**CAR ^1^**	2332.9 ± 91.9 ^a^	1.20 ± 0.06 ^a^	3495.6 ± 66.4 ^b^	1536.8 ± 38.9 ^b^

^1^ GRE1, Grenache; SYR1, Syrah; CAR, Carignan. Data are expressed as the mean of triplicate ± SD. ^2^ ORAC, ABTS^•+^ and DPPH are expressed as µmol Trolox/g Dry Weight (DW) and FRAP as mmol Fe^2+^/g DW. ^a,b,c^; Anova was performed to compare values obtain between varieties for the same test. Same letters indicate no significant differences between the value (Tukey’s test, *p* < 0.05).

**Table 2:** Antioxidant activity characterisation in EAQ and EA70 grape pomace skin extracts.

		ORAC ^2^	FRAP ^2^	ABTS^•+ 2^	DPPH ^2^
**Skins-EAQ**	**GRE2 ^1^**	1190.7 ± 183.6 ^ab^	0.56 ± 0.01 ^c^	934.1 ± 11.9 ^b^	99.5 ± 10.8 ^a^
	**SYR1 ^1^**	1345.9 ± 19.2 ^ab^	0.88 ± 0.01 ^e^	1428,0 ± 54.8 ^c^	690.3 ± 147.0 ^bc^
	**SYR2 ^1^**	1066,0 ± 84.2 ^a^	0.14 ± 0.02 ^a^	668.3 ± 30.0 ^a^	263.9 ± 71.5 ^ab^
	**CAR ^1^**	1077.8 ± 60.2 ^a^	0.67 ± 0.02 ^d^	1048.8 ± 101.6 ^b^	591.0 ± 85.6 ^abc^
	**MOU ^1^**	1033.8 ± 77.6 ^a^	0.32 ± 0.01 ^b^	965.6 ± 16.6 ^b^	279.4 ± 61.7 ^ab^
	**ALI ^1^**	1714.6 ± 14.8 ^b^	1.13 ± 0.00 ^f^	1760.1 ± 91.0 ^d^	1057.1 ± 45.2 ^c^
**Skins-EA70**	**GRE2 ^1^**	1828.3 ± 40.4 ^bc^	1.32 ± 0.03 ^c^	2612.1 ± 130.9 ^a^	877,0 ± 74.3 ^a^
	**SYR1 ^1^**	1912.6 ± 6.1 ^bc^	1.52 ± 0.05 ^d^	2614.5 ± 10.4 ^a^	1391.7 ± 37.2 ^bc^
	**SYR2 ^1^**	1701.8 ± 88.3 ^bc^	0.94 ± 0.03 ^a^	2010.6 ± 147.0 ^a^	1164.9 ± 55.6 ^ab^
	**CAR ^1^**	1238.4 ± 11.1 ^a^	1.34 ± 0.03 ^c^	2555.9 ± 146.0 ^a^	1075.4 ± 46.2 ^ab^
	**MOU ^1^**	2070.0 ± 60.6 ^c^	1.03 ± 0.02 ^ab^	2674.8 ± 187.3 ^a^	833.3 ± 26.4 ^a^
	**ALI ^1^**	1628.5 ± 82.6 ^b^	1.13 ± 0.01 ^b^	1923.4 ± 87.0 ^a^	1749.3 ± 112.7 ^c^

^1^ GRE2, Grenache; SYR1 and SYR2, Syrah; CAR, Carignan; MOU, Mourvèdre; ALI, Alicante. Data are expressed as the mean of triplicate ± SD. ^2^ ORAC, ABTS^•+^ and DPPH are expressed as µmol Trolox/g DW and FRAP as mmol Fe^2+^/g DW. ^a,b,c,d,e,f^; Anova was made to compare values obtain between varieties for the same compound. Same letters indicate no significant differences between the value (Tukey’s test, *p* < 0.05).

**Table 3:** Sum of structurally related urinary metabolites at day 1, 0–8 h and 8–24 h after ingestion of grape pomace extracts. Data are expressed as nmol ± SD.

**Σ** **of Structurally Related Metabolites** **(Day 1, 0–8 h)**	**SHR1**	**SHR5**	**SHR6**	**Control**
(Epi)catechin-*O*-glucuronide	1.0 ± 0.3 *	2.0 ± 0.2 *	1.2 ± 0.5 *	0.1. ± 0.0
*O*-Methyl-(epi)catechin-*O*-glucuronide	8.5 ± 1.5 *	3.5 ± 0.4	6.1 ± 2.7	3.5 ± 1.0
5-(Hydroxyphenyl)-γ-valerolactone-*O*-glucuronide	1.0 ± 0.1 *	1.2 ± 0.4	0.5 ± 0.3	0.4 ± 0.2
5-(Hydroxyphenyl)-γ-valerolactone-*O*-sulphate	1.5 ± 0.3	4.4 ± 2.9	1.2 ± 0.4	1.1 ± 0.3
5-(Hydroxyphenyl)-γ-hydroxyvaleric acid-*O*-sulphate	2.5 ± 0.6 *	3.5 ± 1.3	2.0 ± 0.5 *	0.9 ± 0.3
5-(Phenyl)-γ-hydroxyvaleric acid-*O*-sulphate	6.9 ± 1.3 *	7.6 ± 1.6	6.1 ± 3.7	3.1 ± 0.7
Isoferulic acid-4-*O*-sulfate	157.0 ± 35.5 *	75.0 ± 15.6*	82.8 ± 19.9	82.2 ± 19.9
**Σ** **of Structurally Related Metabolites** **(Day 1, 8–24 h)**	**SHR1**	**SHR5**	**SHR6**	**Control**
(Epi)catechin-*O*-glucuronide	1.1 ± 0.2	1.8 ± 0.6	1.4 ± 0.3	1.0 ± 0.3
O-Methyl-(epi)catechin-*O*-glucuronide	3.8 ± 0.7	6.9 ± 2.1 *	7.1 ± 1.3 *	2.7 ± 1.0
5-(Hydroxyphenyl)-γ-valerolactone-*O*-glucuronide	0.2 ± 0.0	0.4 ± 0.1	0.4 ± 0.1 *	0.2 ± 0.0
5-(Hydroxyphenyl)-γ-valerolactone-*O*-sulphate	1.3 ± 0.2	1.8 ± 0.6	1.6 ± 0.2	1.3 ± 0.3
5-(Hydroxyphenyl)-γ-hydroxyvaleric acid-*O*-sulphate	1.0 ± 0.1	2.6 ± 0.9 *	2.2 ± 0.4 *	0.7 ± 0.1
5-(Phenyl)-γ-hydroxyvaleric acid-*O*-sulphate	12.7 ± 1.8	14.4 ± 2.5	15.8 ± 1.1 *	8.7 ± 0.9
Isoferulic acid-4-*O*-sulfate	80.4 ± 13.6	99.7 ± 16.1	111.5 ± 25.7 *	74.2 ± 4.3

* Metabolites that were excreted in significantly higher amounts compared to their respective control *(p <* 0.05*)*. SHR rats fed with: SHR1, Grenache (GRE1) EA70 seed pomace extract; SHR5, Mourvèdre (MOU) EA70 skin pomace extract and SHR6, Alicante (ALI) EA70 skin pomace extract.

**Table 4:** Sum of structurally related urinary metabolites at day 7, 0–8 h and 8–24 h after ingestion of grape pomace extracts. Data are expressed as nmol ± SD.

**Σ** **of Structurally Related Metabolites** **(Day 7, 0–8 h)**	**SHR1**	**SHR5**	**SHR6**	**Control**
(Epi)catechin-*O*-glucuronide	1.6 ± 1.0	3.1 ± 1.9	2.9 ± 1.0 *	0.5 ± 0.3
*O*-Methyl-(epi)catechin-*O*-glucuronide	5.7 ± 2.2	5.7 ± 2.3	5.9 ± 2.1 *	1.7 ± 0.4
5-(Hydroxyphenyl)-γ-valerolactone-*O*-glucuronide	0.2 ± 0.1	0.3 ± 0.1	0.3 ± 0.1	0.2 ± 0.1
5-(Hydroxyphenyl)-γ-valerolactone-*O*-sulphate	0.2 ± 0.0	0.4 ± 0.2	0.5 ± 0.2	0.2 ± 0.1
5-(Hydroxyphenyl)-γ-hydroxyvaleric acid-*O*-sulphate	0.4 ± 0.1	0.6 ± 0.1 *	0.9 ± 0.4	0.4 ± 0.2
5-(Phenyl)-γ-hydroxyvaleric acid-*O*-sulphate	6.6 ± 1.6	6.7 ± 2.6	8.4 ± 2.3	7.7 ± 2.0
Isoferulic acid-4-*O*-sulfate	131.2 ± 25.9 *	149.2 ± 34.0 *	137.4 ± 45.8	71.8 ± 29.5
**Σ** **of Structurally Related Metabolites** **(Day 7, 8–24 h)**	**SHR1**	**SHR5**	**SHR6**	**Control**
(Epi)catechin-*O*-glucuronide	1.6 ± 0.4	1.6 ± 0.1	2.2 ± 0.4 *	1.1 ± 0.3
*O*-Methyl-(epi)catechin-*O*-glucuronide	8.1 ± 1.8	6.2 ± 1.3	5.2 ± 0.8 *	3.7 ± 0.8
5-(Hydroxyphenyl)-γ-valerolactone-*O*-glucuronide	0.5 ± 0.1	0.7 ± 0.3	0.7 ± 0.1 *	0.4 ± 0.1
5-(Hydroxyphenyl)-γ-valerolactone-*O*-sulphate	0.8 ± 0.1	1.4 ± 0.5	1.6 ± 0.3	1.1 ± 0.3
5-(Hydroxyphenyl)-γ-hydroxyvaleric acid-*O*-sulphate	1.2 ± 0.4	1.8 ± 0.6	1.4 ± 0.3	1.1 ± 0.3
5-(Phenyl)-γ-hydroxyvaleric acid-*O*-sulphate	13.2 ± 4.2	15.4 ± 3.6	14.5 ± 4.0	12.4 ± 2.8
Isoferulic acid-4-*O*-sulfate	209.4 ± 38.6 *	218.2 ± 60.6 *	171.5 ± 43.0	121.2 ± 20.8

* Metabolites that were excreted in significantly higher amounts compared to their respective control *(p <* 0.05*)*. SHR rats fed with: SHR1, Grenache (GRE1) EA70 seed pomace extract; SHR5, Mourvèdre (MOU) EA70 skin pomace extract and SHR6, Alicante (ALI) EA70 skin pomace extract.

**Table 5:** Percentage of intake of different grape pomace extracts excreted in urine at day 1 and day 7, 0–8 h, 8–24 h and 0–24 h after intake.

	Percentage of Intake Day 1 (%)			Percentage of Intake Day 7 (%)		
	SHR1 ^a^	SHR5 ^a^	SHR6 ^a^	SHR1 ^a^	SHR5 ^a^	SHR6 ^a^
**0**–**8 h**	0.83	0.83	0.51	0.94	2.10	1.03
**8**–**24 h**	0.19	0.74	0.51	0.58	2.31	0.39
**Total 24 h**	1.02	1.57	1.02	1.52	4.41	1.43

^a^ SHR rats fed with: SHR1, Grenache (GRE1) EA70 seed pomace extract; SHR5, Mourvèdre (MOU) EA70 skin pomace extract and SHR6, Alicante (ALI) EA70 skin pomace extract.

**Table 6:** Mean plasma metabolite levels at day 7, 4 h after ingestion of grape pomace extracts. Data are expressed as nmol ± SD.

Metabolites	SHR1	SHR5	SHR6
5-(Hydroxyphenyl)-γ-valerolactone-*O*-glucuronide 1	0.62 ± 0.06 *	0.71 ± 0.02	0.46 ± 0.01
(Epi)catechin-*O*-glucuronide 1	Nd	Nd	0.25 ± 0.02 *
(Epi)catechin-*O*-glucuronide 2	0.34 ± 0.01 *	0.32 ± 0.03	0.39 ± 0.03 *
*O*-Methyl-(epi)catechin-*O*-glucuronide 1	0.24 ± 0.02 *	0.17 ± 0.02 *	0.23 ± 0.01 *
5-(Hydroxyphenyl)-γ-valerolactone-*O*-glucuronide 2	0.26 ± 0.01 *	0.31 ± 0.00 *	0.22 ± 0.01 *
Methyl-(epi)catechin-*O*-glucuronide 2	0.62 ± 0.05 *	0.41 ± 0.00	0.71 ± 0.04 *
Methyl-(epi)catechin-*O*-glucuronide 3	Nd	Nd	0.37 ± 0.01 *
Di-methyl-(epi)catechin-*O*-glucuronide 1	0.54 ± 0.06 *	0.86 ± 0.03 *	0.50 ± 0.02 *
5-(Hydroxyphenyl)-4-hydroxyvaleric acid-*O*-sulphate 2	Nd	Nd	Nd
Di-methyl-(epi)catechin-*O*-glucuronide 2	0.42 ± 0.04 *	0.35 ± 0.04 *	0.19 ± 0.01 *
5-(Hydroxyphenyl)-γ-valerolactone sulphate 1	0.42 ± 0.04 *	0.31 ± 0.05 *	0.42 ± 0.06 *

* Metabolites that were excreted in significantly higher amounts compared to their respective control *(p <* 0.05*)*. SHR rats fed with: SHR1, Grenache (GRE1) EA70 seed pomace extract; SHR5, Mourvèdre (MOU) EA70 skin pomace extract and SHR6, Alicante (ALI) EA70 skin pomace extract. SD, standard deviation. Nd, not detected.

**Table 7:** Mean tissue metabolite levels at day 7, after 4 h of grape pomace extracts ingestion. Data are expressed as nmol per organ ± standard deviation.

	Mean Tissue Metabolites Levels (Day 7, after 4 h)	SHR1	SHR5	SHR6
Heart	5-(Hydroxyphenyl)-γ-valerolactone-*O*-glucuronide	2.50 ± 0.25 *	2.64 ± 0.03 *	2.27 ± 0.08 *
	5-(Hydroxyphenyl)-γ-valerolactone-*O*-sulphate	0.11 ± 0.00 *	0.11 ± 0.00 *	Nd
Kidneys	5-(Hydroxyphenyl)-γ-valerolactone-*O*-glucuronide	5.55 ± 0.11	6.62 ± 0.08	5.23 ± 0.34
	5-(Hydroxyphenyl)-γ-valerolactone-*O*-sulphate	0.38 ± 0.00 *	0.37 ± 0.02 *	0.45 ± 0.03 *
Liver	5-(Hydroxyphenyl)-γ-valerolactone-*O*-glucuronide	167.19 ± 8.77	209.98 ± 12.20 *	179.26 ± 0.25
	5-(Hydroxyphenyl)-γ-valerolactone-*O*-sulphate	Nd	Nd	Nd

* Metabolites that were excreted in significantly higher amounts compared to their respective control *(p* < 0.05*)*. SHR rats fed with: SHR1, Grenache (GRE1) EA70 seed pomace extract; SHR5, Mourvèdre (MOU) EA70 skin pomace extract and SHR6, Alicante (ALI) EA70 skin pomace extract. SD, standard deviation. Nd, not detected.
